# Stage III NSCLC Treatment Patterns in Spain: A Population-Based Study of the GOECP-SEOR

**DOI:** 10.3390/cancers17233807

**Published:** 2025-11-27

**Authors:** Paloma Sosa-Fajardo, Margarita Martín-Martín, Javier Luna-Tirado, José Luis López-Guerra, Elías Gomis-Sellés, Miriam López-Mata, Germán Valtueña-Peydró, Marina Santos-Rodríguez, Ana-María Álvarez-González, Guillermo Potdevin-Stein, Paula Martín-Barrientos, Andrea Kannemann, Nuria Farré-Bernadó, Elisabet González-Portillo, Aurea Manso-Lema, Marta Rodríguez-Roldán, María-Ángeles González-Ruiz, Amadeo Wals-Zurita, Nuria-Azahara Linares-Mesa, Sara Garduño-Sánchez, Patricia Barrionuevo-Castillo, Beatriz Moreno-Fuentes, Maximiliano Martos-Alcalde, Paula Simón-Silva, Mariana-Teresa Peña-Perea, Manuel-Luis Blanco-Villar, Victoria Vera-Barragán, Abrahams Ocanto-Martínez, María Mateos-Domínguez, Carmen Vallejo-Ocaña, Raúl Hernanz-Lucas, José-Enrique Castro-Gómez, Marina Peña-Huertas, Víctor Duque-Santana, José Zapatero-Ortuño, Celia García-Torres, Patricia Valencia-Nieto, Patricia Diezhandino-García, Clara Eito-Valdovinos, Alicia Olarte-García, Luis-Leonardo Guerrero-Gómez, Sofía Sánchez-García, Andrea Bobo-Jaureguizar, Amalia Sotoca-Ruiz, Beatriz Vázquez-Barreiro, Guillermo Cameselle-Gallego, Sonia Flamarique-Andueza, Mikel Rico-Oses, Marcos Guijarro-Verdu, Miguel Montijano-Linde, Laura Guzmán-Gómez, Ana-Belén Bezares-Alarcón, Cristina Cigarral-García, Paloma Moreno-Ceano, Javier Serrano-Andreu, Mauricio Murcia-Mejía, María Pagola-Divassón, Francesc Casas-Duran, Piedad Almendros-Blanco, Sara Pedraza-Fernández, José-Antonio González-Ferreira, Pino Alcántara, Felipe Couñago

**Affiliations:** 1 Servicio de Oncología Radioterápica, Hospital Universitario Virgen del Rocío, 41013 Sevilla, Spain; paloma.sosa.sspa@juntadeandalucia.es (P.S.-F.); josel.lopez.guerra.sspa@juntadeandalucia.es (J.L.L.-G.); egomiss@clinic.cat (E.G.-S.); 2 Servicio de Oncología Radioterápica, Hospital Universitario Ramon y Cajal, 28034 Madrid, Spain; margarita.martin@salud.madrid.org (M.M.-M.); mariacarmen.vallejo@slaud.madrid.org (C.V.-O.); raul.hernanz@salud.madrid.org (R.H.-L.); 3 Servicio de Oncología Radioterápica, Fundación Jiménez Díaz, 28040 Madrid, Spain; laura.guzmang@quironsalud.es; 4 Servicio de Oncología Radioterápica, Hospital Clínico Universitario Lozano Blesa, 50009 Zaragoza, Spain; mlopezmat@salud.aragon.es (M.L.-M.); gvaltuenna@salud.aragon.es (G.V.-P.); 5 Servicio de Oncología Radioterápica, Hospital General Universitario Gregorio Marañón, 28007 Madrid, Spain; marina.santos@salud.madrid.org (M.S.-R.); aalvarez@salud.madrid.org (A.-M.Á.-G.); 6 Servicio de Oncología Radioterápica, Hospital Universitario Gran Canaria Doctor Negrín, 35010 Las Palmas de Gran Canaria, Spain; guillermo.potdevin@fvl.org.co (G.P.-S.); pmarbar@gobiernodecanarias.org (P.M.-B.); akannem@gobiernodecanarias.org (A.K.); 7 Servicio de Oncología Radioterápica, Fundación Valle del Lili, 760031 Cali, Colombia; 8 Servicio de Oncología Radioterápica, Hospital Santa Creu i Sant Pau, 08025 Barcelona, Spain; nfarre@santpau.cat; 9 Servicio de Oncología Radioterápica, Hospital Universitario La Paz, 28046 Madrid, Spain; egportillo@salud.madrid.org (E.G.-P.); aurea.manso@salud.madrid.org (A.M.-L.); mrroldan@salud.madrid.org (M.R.-R.); 10 Servicio de Oncología Radioterápica, Hospital Universitario Virgen Macarena, 41071 Sevilla, Spain; mariaa.gonzalez.ruiz.sspa@juntadeandalucia.es (M.-Á.G.-R.); amadeoj.wals.sspa@juntadeandalucia.es (A.W.-Z.); 11 Servicio de Oncología Radioterápica, Hospital Universitario Juan Ramón Jiménez, 21005 Huelva, Spain; nuria.linares.sspa@juntadeandalucia.es (N.-A.L.-M.); sarab.garduno.sspa@juntadeandalucia.es (S.G.-S.); 12 Servicio de Oncología Radioterápica, Complejo Hospitalario de Jaén, 23007 Jaén, Spain; patricia.barrionuevo.sspa@juntadeandalucia.es (P.B.-C.); bmfuentes@salud.madrid.org (B.M.-F.); maximiano.martos.sspa@juntadeandalucia.es (M.M.-A.); 13 Servicio de Oncología Radioterápica, Hospital Universitario Torrecárdenas, 04009 Almería, Spain; paula.simon.sspa@juntadeandalucia.es (P.S.-S.); marianat.pena.sspa@juntadeandalucia.es (M.-T.P.-P.); manuell.blanco.sspa@juntadeandalucia.es (M.-L.B.-V.); 14 Servicio de Oncología Radioterápica, Hospital Universitario de Badajoz, 06006 Badajoz, Spain; victoria.vera@salud-juntaex.es; 15 Servicio de Oncología Radioterápica, Hospital Universitario de Torrejón, 28850 Madrid, Spain; abrahams.ocanto@genesiscare.es (A.O.-M.); maria.mateos@genesiscare.es (M.M.-D.); 16 Servicio de Oncología Radioterápica, Complejo Hospitalario Universitario de Orense, 32005 Orense, Spain; jose.enrique.castro.gomez@sergas.es; 17 Servicio de Oncología Radioterápica, QuirónSalud, 28036 Madrid, Spain; marina.pena@quironsalud.es (M.P.-H.); victor.duque.co@quironsalud.es (V.D.-S.); 18 Departamento de Medicina, Facultad de Ciencias Biomédicas, Universidad Europea, 28670 Madrid, Spain; 19 Servicio de Oncología Radioterápica, Hospital Universitario Rey Juan Carlos, 28933 Madrid, Spain; jose.ortuno@hospitalreyjuancarlos.es (J.Z.-O.); celia.garciat@hospitalreyjuancarlos.es (C.G.-T.); 20 Servicio de Oncología Radioterápica, Hospital Clínico Universitario de Valladolid, 47003 Valladolid, Spain; pvalencia@saludcastillayleon.es (P.V.-N.); pdiezhandino@saludcastillayleon.es (P.D.-G.); 21 Servicio de Oncología Radioterápica, Clínica IMQ Zorrotzaurre, 48014 Bilbao, Spain; c.eito@imq.es (C.E.-V.); a.olarte@imq.es (A.O.-G.); 22 Servicio de Oncología Radioterápica, Hospital La Luz, 28041 Madrid, Spain; luis.guerrerog@quironsalud.es (L.-L.G.-G.); sofia.sanchez@quironsalud.es (S.S.-G.); 23 Servicio de Oncología Radioterápica, Hospital Ruber Internacional, 28034 Madrid, Spain; andrea.bobo@ruberinternacional.es (A.B.-J.); asotoca@ruberinternacional.es (A.S.-R.); 24 Servicio de Oncología Radioterápica, Hospital Meixoeiro, 36214 Vigo, Spain; beatriz.vazquez.barreiro@sergas.es (B.V.-B.); guillermo.cameselle.gallego@sergas.es (G.C.-G.); 25 Servicio de Oncología Radioterápica, Hospital Universitario de Navarra, 31008 Navarra, Spain; sonia.flamarique.andueza@navarra.es (S.F.-A.); mikel.rico.oses@cfnavarra.es (M.R.-O.); 26 Servicio de Oncología Radioterápica, GenesisCare San Francisco de Asís, 28002 Madrid, Spain; marcos.guijarro@genesiscare.es (M.G.-V.); m.montijano@imsan.aw (M.M.-L.); 27 Servicio de Oncología Radioterápica, GenesisCare Campo de Gibraltar, 11207 Algeciras, Spain; anabelen.bezares@genesiscare.es; 28 Servicio de Oncología Radioterápica, Hospital Clínico de Salamanca, 37005 Salamanca, Spain; ccigarral@saludcastillayleon.es; 29 Servicio de Oncología Radioterápica, GenesisCare Málaga, 29010 Málaga, Spain; paloma.moreno@genesiscare.es; 30 Servicio de Oncología Radioterápica, Clínica Universidad de Navarra, 28027 Madrid, Spain; fserranoa@unav.es; 31 Servicio de Oncología Radioterápica, Hospital Universitari Sant Joan de Reus, 43204 Barcelona, Spain; mauricio.murcia@salutsanjoan.cat; 32 Servicio de Oncología Radioterápica, Hospital Universitario Fundación Onkologikoa, 20014 Donostia, Spain; mpagola@onkologikoa.org; 33 Servicio de Oncología Radioterápica, Hospital Clinic Barcelona, 08036 Barcelona, Spain; fcasas@clinic.cat; 34 Servicio de Oncología Radioterápica, Hospital General Universitario de Valencia, 46014 Valencia, Spain; almendros_pie@gva.es; 35 Servicio de Oncología Radioterápica, Hospital 12 de Octubre, 28041 Madrid, Spain; sara.pedraza@salud.madrid.org; 36 Servicio de Oncología Radioterápica, GenesisCare, 41092 Sevilla, Spain; jose.gonzalez@genesiscare.es; 37 Servicio de Oncología Radioterápica, Hospital Clínico de San Carlos, 28040 Madrid, Spain; mariapino.alcantara@salud.madrid.org; 38 Servicio de Oncología Radioterápica, GenesisCare Madrid, Hospital San Francisco de Asís and Vithas La Milagrosa, Universidad Europea de Madrid, 28002 Madrid, Spain; felipe.counago@genesiscare.es

**Keywords:** non-small cell lung cancer stage III, concurrent chemoradiotherapy, immunotherapy, durvalumab, observational, population-based, real-world study

## Abstract

**Objective:** We aimed to provide real-world insights into the management of non-small cell lung cancer stage III (NSCLC-SIII) in Spain. **Methods:** The GOECP-SEOR group conducted an observational, retrospective, multicenter study in which data from patients diagnosed with NSCLC-SIII treated in Spanish radiotherapy departments (RTDs) were collected. **Results:** A total of 1505 NSCLC-SIII patients from 35 RTDs from 2018 to 2021 were recruited. A total of 871 patients (57.9%) received concurrent chemoradiotherapy (cCRT), and 390 patients (25.91%) received maintenance durvalumab. The immunotherapy (IT) was well tolerated, with Common Terminology Criteria for Adverse Events (CTCAE) Grade 0–2 toxicity observed in 85% of the patients (295 patients). The median overall survival (OS) was 26 months (95% CI 9.4-Not Reached NR). There was a statistically significant difference when patients were stratified by IT, with a median OS of 40 months (95% CI 22.1-NR) in the IT maintenance cohort compared with 19.4 months (95% CI 7.1–58.1) in the non-IT cohort. Differences were also noted in progression-free survival (PFS), with a median PFS of 20.8 months (95% CI 9.6-NR) in the IT cohort versus 8.4 months (95% CI 3.1–25.1) in the non-IT cohort. These differences remained significant according to the multivariate model (OS: HR 0.45 [95% CI 0.36–0.56], *p* < 0.001; PFS: HR 0.5 [95% CI 0.42–0.59], *p* < 0.001). **Conclusions:** The management of NSCLC-SIII patients can vary, leading to differences in treatment outcomes. The treatment of lung cancer in NSCLC-SIII patients in RTDs across Spain aligns with international guidelines and expert recommendations.

## Simple Summary

This study analyzed real-world treatment patterns for stage III non-small cell lung cancer (NSCLC-SIII) in Spain. Data from 1505 patients treated at 35 radiotherapy departments between 2018 and 2021 were reviewed. Of these, 57.9% received concurrent chemoradiotherapy (cCRT), and 25.9% received maintenance immunotherapy with durvalumab. Immunotherapy was well tolerated in most patients. Those who received maintenance immunotherapy had significantly better outcomes, with a median overall survival of 40 months compared to 19.4 months in those who did not receive it. Progression-free survival was also longer in the immunotherapy group (20.8 vs. 8.4 months). These benefits remained significant after adjusting for other factors. The findings confirm that Spanish treatment practices largely align with international standards and that maintenance immunotherapy improves survival in NSCLC-SIII.

## 1. Introduction

### 1.1. Background

NSCLC-SIII encompasses a broad spectrum of tumors with heterogeneous development, prognoses, and treatment possibilities. Trimodal treatment with surgery, radiotherapy, and chemotherapy in various combinations and sequences is the most common and beneficial, as reflected in important international consensus and guidelines [1,2,3]. Traditionally, the standard treatment for advanced and unresectable tumors was cCRT for 6 weeks at a dose of 60 Gy in 30 daily fractions of 2 Gy plus platinum-doublet-based chemotherapy. However, the oncological outcomes were far from ideal, with poor survival rates [4]. Several attempts have been made over the years to improve OS, either by continuing chemotherapy, using other systemic therapies, or escalating radiation doses, but all of these attempts have failed [4,5,6,7,8,9,10,11,12,13,14]. As a result of these disappointing results, the standard management of locally advanced NSCLC remained unchanged for 15 years until the publication of the PACIFIC study [15,16,17], where 713 patients diagnosed with NSCLC-SIII (AJCC v7) and treated with cCRT were randomized after concomitant chemoradiotherapy to receive maintenance therapy with intravenous durvalumab vs. placebo. PFS and OS were calculated from randomization, and little information about radiotherapy characteristics was published. The study revealed a statistically significant difference in the median PFS between arm A (durvalumab) (16.8 months; 95% confidence interval [CI] 13–18.1) and arm B (placebo) (5.6 months; 95% CI 4.8), with a hazard ratio (HR) of 0.52, (95% CI 0.42–0.65, *p* < 0.001). This difference was maintained for PFS-12 months and PFS-18 months. A later update from the PACIFIC study was presented by Spiegel et al. [18]: after 5 years of follow-up, the benefit in OS and PFS with durvalumab remained robust (42.9% still alive and 33.1% had no disease vs. 33.4% and 19%, HR 0.72; 95% CI 0.59–0.89). Following these results, the use of maintenance therapy with durvalumab has expanded throughout our community, with numerous centers already publishing their experience with the use of this drug [19,20,21,22].

The benefit of immunotherapy in lung cancer has been previously confirmed in several studies, demonstrating a clear correlation between PDL1 overexpression and immunotherapy efficacy. In Keynote 001 [23], a clear correlation was observed between tumor PD-L1 expression level (TPS) and response to pembrolizumab, in terms of objective response rate (ORR), progression-free survival (PFS), and overall survival (OS). Among patients with PD-L1 TPS ≥ 50%, the ORR was 51.9% (14/27; 95% CI: 32–71%) compared with 27% in the overall study population. The median PFS was 6.2 months (95% CI: 4.1–8.6) in the total population, whereas in the subgroup with PD-L1 TPS ≥ 50% it reached 12.5 months (95% CI: 22.1 to not reached).

However, data published from several European groups, such as the British [3] and Dutch [24] groups, suggest that, owing to various logistical difficulties, access to radiotherapy centers, etc., cCRT, despite being the gold standard, is not always the most common treatment for patients. An observational French study [25] examined patient profiles and clinical outcomes in the treatment of unresectable NSCLC-SIII. Patients treated with concurrent chemoradiotherapy had higher 12- and 24-month survival rates, emphasizing the importance of optimized patient selection. Therefore, the Oncology Group for the Study of Lung Cancer (GOECP) proposed conducting the first retrospective, multicenter population study that provides “real-world data” on the management of NSCLC-SIII carried out in Spanish RTDs. The overarching goal of the proposed multicenter population study by the GOECP is to obtain “real world” data on the treatment received by patients with NSCLC-SIII in Spanish RTDs. This involves understanding the scope and real accessibility of various treatments for Spanish patients.

### 1.2. Problem Statement

Real-world treatment practices for stage III non–small cell lung cancer (NSCLC-SIII) in Spanish Radiation Therapy Departments remain unclear, and clinical trial data do not fully represent routine care. To address this gap, the GOECP proposes a retrospective, multicenter study to describe the actual treatment patterns and access to therapies in this population.

## 2. Materials and Methods

### 2.1. Design

This retrospective multicenter observational study aimed to obtain real-world data on the treatment of NSCLC-SIII patients in Spanish RTDs. Since the Spanish Agency of Medicines issued a favorable therapeutic positioning report for the use of durvalumab as maintenance therapy in the PACIFIC setting in January 2020, the study included patients who could provide data from both the pre- and post-PACIFIC eras. Therefore, data were collected from patients treated between 2018 and 2022.

Notably, in Spain, patients with PD-L1 < 1% are not allowed to be treated with immunotherapy as a standard. This multicenter study was endorsed by the Lung Cancer Study Group of the Spanish Society of Radiation Oncology (GOECP/SEOR). This study was approved by the ethics committee of investigation Fundación Jiménez Díaz, Madrid (Study code: EO018-22_HUQM, date: 18 January 2022). Since the data were collected retrospectively following real-world clinical practice, no additional informed consent was required.

The aim of this study is to identify real-world treatment patterns for patients with NSCLC-SIII in Spanish RTDs.

#### Specific Objectives

1. To characterize the management approaches used for NSCLC-SIII, including radiotherapy, chemotherapy, and consolidation strategies.
2. To assess the accessibility of Durvalumab maintenance.

### 2.2. Selection of Patients

The inclusion criteria were patients with a clinical and histological diagnosis of NSCLC-SIII (AJCC 8th edition) treated in a Spanish RTD, who received radiotherapy with neoadjuvant, adjuvant, radical, or palliative intent, and with acceptance of trimodal treatment combinations (surgery-chemotherapy-radiotherapy) in various modalities.

Patients with a small cell lung tumor, tumors without histological confirmation, or tumors not classified as Stage III according to the AJCC version 8 were excluded.

### 2.3. Data Collection

Epidemiological data, treatment information, follow-up data, disease status, survival, and toxicity data were collected. Patients were treated consecutively at each center, and their data were recorded until death or loss to follow-up, capturing subsequent treatment lines and new events. Concomitant chemoradiotherapy was defined the same as that of the PACIFIC trial, with at least 2 platinum-based cycles during radiotherapy. Finally, the real-world results from our study were compared with those from population studies in neighboring countries and international clinical guidelines.

The study data were collected using an electronic REDCap database hosted at IRYCIS. REDCap is a secure web application designed to support data capture in research studies. The database was designed in compliance with data protection regulations, including the EU General Data Protection Regulation (GDPR) and the Organic Law 3/2018 of Data Protection and Digital Rights Guarantee. Security measures for data transmission also comply with R.D.1270/2007.

### 2.4. Ethics Committee Approval

All eligible centers received the approval of an ethics committee.

### 2.5. Statistical Analysis

A total of 1505 patients treated nationwide were included, with collaboration from 35 Spanish RTDs. Patients included in the study were treated between 1 January 2018 and 31 December 2022, and the follow-up lasted until 23 February 2023.

Descriptive statistics are presented as medians (interquartile ranges) or means ± standard deviations for continuous variables and absolute or relative frequencies for categorical variables. Chi-square tests and Student’s *t* tests were used to analyze significant differences between characteristics based on whether surgery was performed. Nonparametric tests (Mann–Whitney U) were used if normal distribution conditions were not met, maintaining a significance level of *p* < 0.05 for all tests. Survival and disease-free survival were evaluated using the Kaplan–Meier method. The OS of the included patients was calculated considering the end date of radiotherapy as the origin of follow-up and the day of death or last follow-up as the final date and PFS considering the end date of radiotherapy as the origin of follow-up and the day of progression established locally, death, or last follow-up as the final date. Univariate analysis using the log-rank test was conducted to identify individual relationships. Multivariate analysis using Cox regression was subsequently performed, incorporating variables with significant univariate analysis results (significance level *p* < 0.05). All analyses were performed with Stata/BE v17.0.

## 3. Results

### 3.1. Baseline Characteristics of Patients and Disease

A total of 1505 patients diagnosed with NSCLC-SIII from 35 Spanish centers were included in the analysis. The demographic data are presented in Table 1. The mean age was 67 years, with 76% of the patients being males and 93% being active or former smokers, with an ECOG of 0 in 52.2% of the patients. Stage IIIA disease was present in 44.2% of the patients, and stage IIIB disease was present in 43.7%. The most common histology was squamous cell carcinoma, accounting for 47.2% of the cases. Among the whole sample, 2.1% presented with EGFR mutations, 1.1% presented with ALK mutations, and 0.35% presented with ROS-1 mutations. PD-L1 status was obtained for 56% of patients, 73.2% of whom had a PD-L1 > 1.

### 3.2. Treatment Characteristics

**Figure 1:**
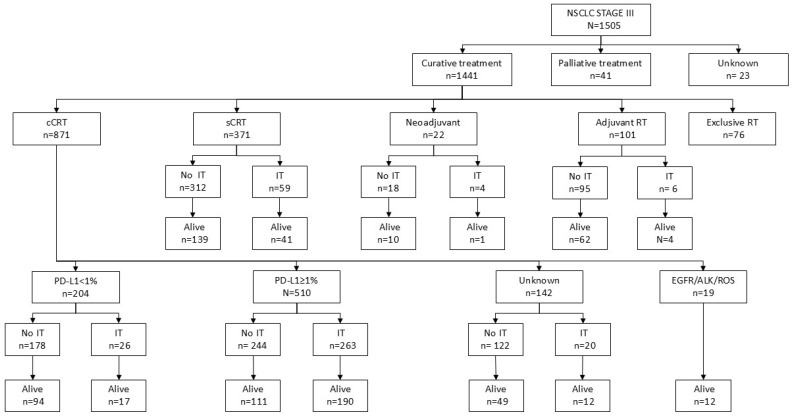
Flow chart of the results by type of treatment. cCRT, concomitant chemoradiotherapy; IT, immunotherapy; NSCLC, non-small cell lung cancer; sCRT, secondary chemoradiotherapy; RT, radiotherapy.

The treatment characteristics are presented in Table 2, and Figure 1 shows a flowchart of the results by type of treatment. The most commonly used treatment was concurrent chemoradiotherapy in 58% of patients, followed by sequential treatment in 24.7%. The average radiation therapy dose was 60 Gy, and the intensity-modulated radiotherapy (IMRT) technique was used in 77.8% of the patients. The most common chemotherapy regimen was the platinum doublet (73.6%). Surgery was performed in 10.49% of the patients, with lobectomy being the most utilized procedure (8.4%). Surgery was planned both as definitive treatment in trimodal therapy and as salvage treatment after RTQT with radical intent. Immunotherapy was administered to 26% of the patients, with durvalumab, according to the PACIFIC regimen, 80% after concurrent CRT and 15.2% after sequential chemoradiotherapy. The average duration of immunotherapy maintenance was 9.3 months. Of the 390 patients in the cohort who received immunotherapy, 121 received it during the pre-PACIFIC period (2018–2019), and 269 during the post-PACIFIC period (2020–2022).

### 3.3. Local Control

A total of 52.7% of patients who received concurrent chemoradiotherapy experienced some form of recurrence. Among them, 44% were distant, 39% were local, and 17% were both local and distant. The most common site of metastasis was the central nervous system (46.5%), followed by bone (40%) and lungs (33.6%). This pattern of recurrence was comparable between the cohort that received immunotherapy and the cohort that did not. (Table 3).

### 3.4. Overall Survival

**Figure 2:**
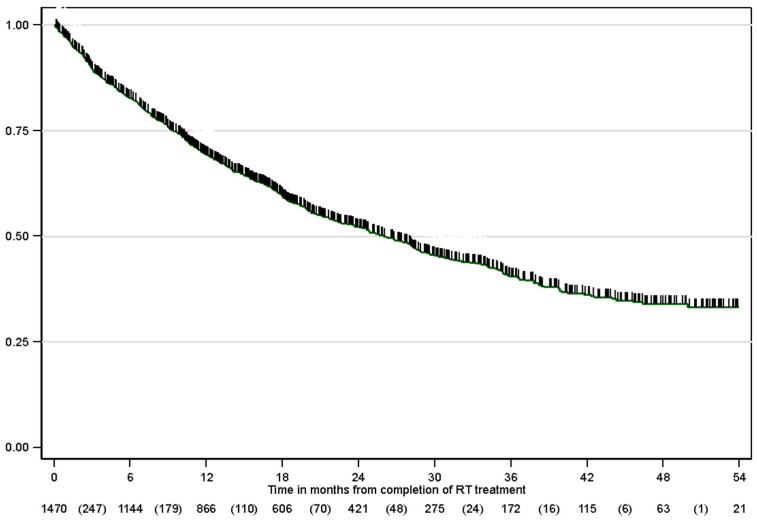
Overall survival in the whole population.

**Figure 3:**
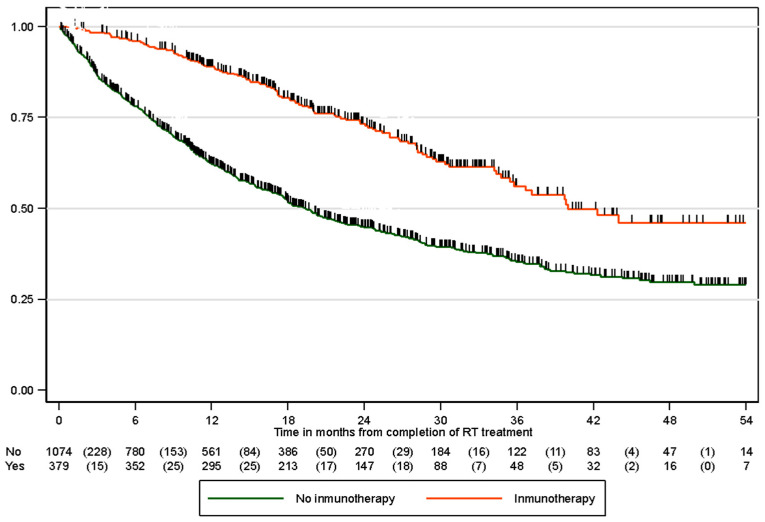
Overall survival according to immunotherapy.

Among the 1505 included patients, 702 (46.6%) died. The estimated median overall survival by the Kaplan–Meier method for the entire group was 26 months (95% CI 9.4–NA) (Figure 2). In total, 871 patients were treated with radical intent cCRT. For this group, the median survival of patients treated with immunotherapy was 43.9 months (95% CI 21.7–NA months), whereas that of patients without immunotherapy was 19.4 months (95% CI 7.4–58.1 months) (*p* < 0.001) (Figure 3). Regarding PD-L1 status, there was no statistically significant difference between the groups, although there appeared to be a more favorable trend in the group with PD-L1 overexpression > 50% than in the 1–49% group (95% CI not reached vs. 43 months). Fifty-one patients who were treated with CRT and whose PD-L1 status was unknown received immunotherapy according to the PACIFIC schedule. Since this was a retrospective study, these data were probably not collected from the database in most of them, and PD-L1 was probably greater than or equal to 1%, although a minority of them could receive immunotherapy treatment within the clinical trial. The survival rates at 6, 12, and 24 months in the subgroup of patients who received immunotherapy are shown in Table 4. In the univariable study, immunotherapy (HR 0.42, 95% CI 0.35–0.52), age, female sex, stage IIIA-B disease, adenocarcinoma histology and lack of comorbidities were predictive factors for improved overall survival. In the multivariable study, immunotherapy treatment remained a significant factor for improved survival (HR 0.42, 95% CI 0.34–0.52), adjusted for sex, stage, histology, and respiratory and cardiovascular comorbidities (Table 5).

### 3.5. Progression-Free Survival

**Figure 4:**
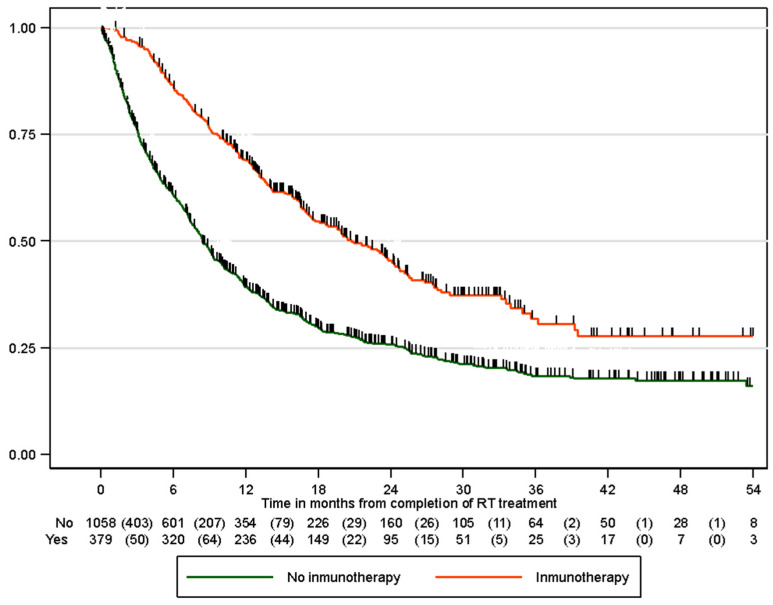
Progression-free survival according to immunotherapy status.

A total of 974 (64.7%) patients experienced disease progression, with a median time to progression of 11.2 months (95% CI 4.2–31.9 months). For patients treated with radical intent cCRT who did not receive immunotherapy, the median time to progression was 8.4 months (95% CI 3.1–25.1 months), whereas for patients who received immunotherapy, it was 20.8 months (95% CI 8.6–NA months) (*p* < 0.001) (Figure 4). PFS was better in patients with PD-L1 > 50% than in those with PD-L1 ranging from 1 to 49% (35 vs. 19 months, *p* < 0.001). The probabilities of disease-free survival at 6, 12, and 24 months, depending on whether the patients received immunotherapy, are shown in Table 4. In the univariable analysis, immunotherapy (HR 0.51 95% CI 0.44–0.59; *p* < 0.001), younger age, male sex, advanced early-stage disease, adenocarcinoma histology and lack of vascular and respiratory comorbidities were predictive factors for better disease-free survival (Table 6). In the multivariable study, immunotherapy treatment remained a predictive factor for better disease-free survival (HR 0.48, 95% CI 0.41–0.57), adjusted for age, sex, stage, histology, and pulmonary and cardiac comorbidities (Table 6).

### 3.6. Toxicity

The incidence of Grade 3 or higher toxicity was 13.8% in the overall group, with an overall incidence of pneumonitis greater than Grade 2 of 3.2%. Among patients treated with concomitant chemoradiotherapy, the incidence of pneumonitis greater than Grade 2 was 3.7%, the incidence was lower among patients who did not receive immunotherapy *n* (1.6%), than among those who received immunotherapy (6.15%). The incidence of esophagitis in patients who received concomitant CRT was 5.7%, regardless of whether they received immunotherapy. Other types of toxicity in patients treated with immunotherapy are shown in Table 7.

## 4. Discussion

NSCLC-SIII is one of the most complex scenarios in oncology overall. The appropriate choice of the best treatment is a significant challenge and is dependent on multiple factors. First, it relies on accurate staging and clinical assessment of the patient to determine the best course of treatment. Second, the case of a tumor committee that makes the final decision on treatment, which is based on the patient’s clinical and functional status, tumor resectability, number of affected or suspicious lymph nodes and nodal regions, and molecular factors (PDL1, EGFR, ALK, etc.), among other factors, should be discussed. NSCLC-SIII is a tremendously dynamic scenario in which scientific studies and available evidence concerning different disease settings exist. In resectable patients, the advent of chemoimmunotherapy has revolutionized neoadjuvant therapy, limiting the indications for radiotherapy, at least for the time being, in this setting [26]. In terms of postoperative management, the results of the LUNG-ART trial have restricted the indications for radiotherapy, in general, to patients with incomplete resections and transcapsular nodal involvement [27]. With respect to unresectable/inoperable NSCLC-SIII, the results of the PACIFIC trial [15,17,22] have drastically changed the approach, making cCRT with sequential durvalumab for 12 months the standard treatment, with a significant improvement in OS and PFS, without increased toxicity compared with classic radiochemotherapy. Since phase III randomized studies may tend to overrepresent patients with good ECOG and younger ages, the results must be verified in less selected populations, with truly important studies such as the PACIFIC-R study [28,29], which retrospectively evaluated data from 1399 patients treated between September 2017 and December 2018, confirming the excellent results of the PACIFIC trial without a significant increase in toxicity in the elderly population. In line with this, the results of a retrospective U.S. study [30] involving 528 patients diagnosed with unresectable NSCLC-SIII demonstrated that maintenance with durvalumab after cCRT in a real-world setting preserves the overall survival benefit observed in the phase III PACIFIC trial. Another study, which was based on data from the Netherlands Cancer Registry [31], analyzed the use of durvalumab after chemoradiotherapy in patients with stage III NSCLC. The results show that overall survival in clinical practice is comparable to trial outcomes, with a 4-year survival rate of 53%. Patients with negative PD-L1 expression had similar outcomes to those with positive PD-L1 expression 1–49, which calls into question current regulatory restrictions. Additionally, a recent update of the PACIFIC-R study published in ESMO 2024 [32] reported a 3-year overall survival rate of 63.2%. Outcomes were better in patients with positive PD-L1 expression (≥1%) and those receiving concurrent CRT, although favorable results were also noted in patients without PD-L1 expression (<1%) and those undergoing sequential CRT. In this changing scenario, the GOECP conducted this study to determine the usual practice in different stage III scenarios in the RTDs of Spain. Thirty-six centers located in 11 of the 19 autonomous communities in Spain participated, recruiting patients treated from 2018 to 2020. Therefore, this study provides relevant information regarding the management of stage III NSCLC in the era of the initiation of immunotherapy in this setting following the results of the PACIFIC trial [17,22]. To the best of our knowledge, including a total of 1505 patients, the present study is one of the most comprehensive population studies on NSCLC-SIII to date. Unlike the Dutch study [24], where all the localized stages (I-III) were analyzed and aspects related to the use of immunotherapy were not included, the GOECP study analyzed only the characteristics and type of treatment of stage III NSCLC, with 25.9% of patients receiving treatment with durvalumab. Additionally, in 2019, the British group published changes in the NSCLC treatment landscape in Great Britain over the past 20 years [33]. These changes began in 2004 with the establishment of a national database, the National Lung Cancer Audit (NLCA). The creation of the NLCA was prompted by the need to standardize the management of this pathology upon realizing that the oncological outcomes of British patients with NSCLC were worse than those reported by other countries. This database was also used for the study published in 2019 by Adizie et al. [3], which analyzed data on the treatment of stage III NSCLC in patients treated during 2016 in England. The results revealed that in Great Britain, only 17% of patients were treated with radical radiotherapy (RT). In contrast, in our study, only 20% of patients received palliative radiotherapy, with 11% also receiving chemotherapy (CT). In the British study, the 1-year survival rate was 32.9% (37.4% for stage IIIA patients), whereas in our study, it was 67.6% (70.1% for stage IIIA patients). The authors concluded that this analysis underscores the importance of multimodal treatment and optimal, standardized patient management. Regarding treatment characteristics, a significant point from our population study is the description of the type of radiotherapy used. Various studies have demonstrated the superiority of IMRT over 3D radiotherapy [34]. In this study, 1171 patients (77.8%) received IMRT, with image-guided control (IGRT with CBCT) performed in 87.4% of all patients, demonstrating the high technological level in the analyzed period in the treatment of radiotherapy for lung cancer in Spanish Radiation Oncology. With a mean dose of 60.2 Gy, other data that highlight the quality of treatments include the total radiotherapy time, with an average of 44.0 days (95% CI 40.0–49.0). One of the aspects that can generate more debate is the percentage of patients receiving concurrent radiochemotherapy (58%) and sequential chemotherapy and radiotherapy (24.7%). The objective of this study was not to determine why these figures exist, but undoubtedly, the percentage of patients receiving concomitant treatment is high, reflecting a possible patient selection bias inherent to the retrospective nature of this study. The reasons for choosing a sequential regimen are usually diverse, depending on the scenario of each hospital or health care service, in addition to the patient’s clinical situation, but the determination and analysis of these causes are the subject of other studies. Surgery was administered to 20% of stage IIIA patients, half of whom also underwent adjuvant CT, predominantly. Other noteworthy data, in the surgical scenario, include the decreasing role of radiotherapy both in the neoadjuvant (1.5% of study patients) and adjuvant settings (6.7%, incomplete resections or N2). With the inclusion of IT-chemotherapy combinations in the neoadjuvant setting [26], new treatment selection algorithms need to be taken into account.

Our real-world study population was largely divided into two subgroups: patients who received immunotherapy according to the PACIFIC scheme (26%) and those who did not. There were no statistically significant differences in demographic terms between the two groups. The characteristics of the group treated with immunotherapy are similar to those of the patients in the PACIFIC-R study, with a mean age of 65 years in our study vs. 66 years in the PACIFIC-R study; female sex (27.7% vs. 32.5%); former smoker (49% vs. 59.5%); ECOG 0 (61.6% vs. 51.4%); nonsquamous carcinoma (52.1% vs. 63.1%); and stage IIIB/IIIC (60.5% vs. 51%). The PD-L1 status was known in 56% of patients, a very good figure considering that these patients were assessed between 2018 and 2020, a period in which such determination was not implemented in the same way in all hospitals and health systems that make up the Spanish National Health System. Eighty percent of these patients treated with IT had previously received cCRT treatment. The mean duration of IT treatment was 9.3 months. Other real-world studies reported similar data: PFS was longer in the PACIFIC-R study [28] than in its equivalent arm in the PACIFIC study [17]; the median PFS was 21.7 months (95% CI, 19.2–24.5) in the entire cohort of the PACIFIC-R study (*n* = 1399) compared with 16.9 months (95% CI, 13–23.9) in the durvalumab arm of the PACIFIC study (*n* = 476). Girard and his team [29] noted that real-world PFS might be overestimated because of methodological differences between the studies. In our study, the PFS was 20.8 months for patients treated with cCRT and durvalumab, confirming improved results with IT compared with the PACIFIC trial in real-world studies. Our study also confirmed better outcomes with higher PD-L1 expression. In terms of OS, our results are similar to those of the PACIFIC trial (43.9 months vs. 47.5 months, respectively), with no significant differences observed depending on the level of PD-L1 expression. Owing to population heterogeneity and differences in the statistical analyses performed, direct comparisons cannot be made. Nevertheless, they undeniably reflect the positive practices and outcomes observed in our study. The importance of immunotherapy treatment is also highlighted in this study through the multivariable analysis of both OS and PFS. In both cases, immunotherapy treatment was shown to be a predictive factor. During the analyzed period, 52.7% of the 871 patients who received cCRT experienced some form of recurrence. Among them, 61% of patients experienced systemic progression of the disease, while the rate of local recurrence/progression within the radiotherapy volume was 19.8%, which was slightly higher than the rate of recurrence described in the R-world study, which reported a value of 10.3%. The percentage of brain metastases was low in patients treated with immunotherapy, standing at 8.9% in our study. Finally, the toxicity recorded in this study was notably positive, with an overall incidence of Grade 3 or higher toxicity of 16.3% in the overall group. There were low rates of severe esophagitis (4.1%) and severe pneumonitis (6.9%). This incidence of pneumonitis is lower than that reported in other studies, such as most of the studies included in the systematic literature review and meta-analysis by Kuang et al. [35]. However, all the authors agreed that cCRT and immunotherapy, in addition to providing significant advantages in terms of OS and PFS, represent safe and well-tolerated treatment options.

## 5. Future Perspectives and Recommendations

Future perspectives in the field of radiotherapy for locally advanced lung cancer involve further optimization of radiotherapy combinations with different systemic treatments. Radiotherapy has a well-recognized immunostimulatory potential and is capable of inducing responses not only in the treated lesion but also at distant tumor sites. However, many aspects regarding potential combinations, optimal dosing, sequencing, and timing remain to be clarified. However, recent approval of neoadjuvant chemo-immunotherapy followed by surgery for potentially resectable stage III tumors has opened a new therapeutic option for this patient profile. This development highlights the need for multidisciplinary tumor boards to individualize treatment decisions and to assess the potential resectability of each lesion. The future challenge lies in refining the definition of “potentially resectable” and establishing clinical trials to directly compare both therapeutic approaches in this setting.

## 6. Study Limitations

Our study has several limitations. First, owing to its observational design, the comparison between treatment modalities is subject to bias, especially confusion by indication; although we performed a multivariate analysis, residual confounding is likely to be present. Second, this study offers a snapshot of routine practice in the management of stage III non-small cell lung cancer in Spain between 2018 and 2021. We are aware that numerous advances in this field mean that routine practice is constantly evolving, with new molecular information and treatments being introduced

## 7. Conclusions

In conclusion, our study provides valuable real-world insights into the management of stage III non-small cell lung cancer in Spain. Our findings underscore the alignment of treatment practices in Spanish RTD with international guidelines and expert recommendations. Notably, adherence to concurrent chemoradiotherapy (cCRT) stands out, with over 57% of patients receiving this regimen, reflecting an improvement over previous data. Furthermore, the oncological outcomes observed in our study are consistent with the literature, emphasizing the efficacy and tolerability of current treatment modalities. Notably, maintenance IT with durvalumab following the PACIFIC schedule significantly improved both OS and PFS compared with those in non-IT cohorts. Age and tumor stage emerged as significant prognostic factors, highlighting the importance of personalized treatment approaches. Our results highlight the importance of having complete molecular information about lung cancer in each case to select the best oncological treatment, as well as the relevance of proper coordination among the medical teams involved. Furthermore, it is essential to continue the ongoing research and innovation in the field of lung cancer treatment.

## Figures and Tables

**Table 1:** Demographic and clinical characteristics.

	Total *N* = 1505	No Immunotherapy *N* = 1115	Immunotherapy * N = 390	
Age at diagnosis, mean (SD)	67.1 (9.1)	67.8 (9.3)	65.1 (8.1)	0.411
Gender, *n* (%)				0.54
Female	358 (24.1)	251 (22.8)	107 (27.7)	
Male	1127 (75.9)	848 (77.2)	279 (72.3)	
Performance Status, *n* (%)				<0.001
0	754 (52.2)	526 (48.9)	228 (61.6)	
1	541 (37.4)	421 (39.2)	120 (32.4)	
2	110 (7.6)	96 (8.9)	14 (3.8)	
3	8 (0.6)	8 (0.7)	0 (0.0)	
Missing	32 (2.2)	24 (2.2)	8 (2.2)	
Smoking status, *n* (%)				0.039
Active smoker	575 (38.3)	410 (36.8)	165 (42.5)	
Former smoker	820 (54.6)	630 (56.6)	190 (49.0)	
Never smoker	81 (5.4)	58 (5.2)	23 (5.9)	
Missing	25 (1.7)	15 (1.3)	10 (2.6)	
Comorbidities, *n* (%)				0.04
Cardiovascular	706 (46.9)	542 (48.6)	164 (42.1)	
Respiratory	508 (33.8)	406 (36.4)	102 (26.2)	
Metabolic	524 (34.8)	409 (36.7)	115 (29.5)	
Previous tumors	234 (15.5)	179 (16.1)	55 (14.1)	
Other	310 (20.6)	244 (21.9)	66 (16.9)	
Histology, *n* (%)				0.14
Epidermoid	711 (47.2)	524 (47.0)	187 (47.9)	
Adenocarcinoma	644 (42.8)	466 (41.8)	178 (45.6)	
Carcinoma	67 (4.5)	55 (4.9)	12 (3.1)	
Large cells	40 (2.7)	35 (3.1)	5 (1.3)	
Others	18 (1.2)	15 (1.3)	3 (0.8)	
Missing	25 (1.7)	20 (1.8)	5 (1.3)	
Mutations, *n* (%)				<0.001
Unknown	316 (21.0)	288 (25.8)	28 (7.2)	
PD-L1 Unknown	652 (43.3)	586 (52.6)	66 (16.9)	
PDL1 < 1%	228 (15.1)	228 (20.4)	-	
PDL1 > 1–50%	383 (25.4)	192 (17.2)	191 (49)	
PDL1 > 50%	242 (16.1)	109 (9.8)	133 (34.1)	
EGFR	32 (2.1)	27 (2.4)	5 (1.3)	
ALK	16 (1.1)	15 (1.3)	1 (0.3)	
ROS1	5 (0.3)	5 (0.4)	0 (0.0)	
T, *n* (%)				0.191
Tx	28 (1.9)	17 (1.5)	11 (2.8)	
T0	1 (0.1)	1 (0.1)	0 (0.0)	
Tis	1 (0.1)	1 (0.1)	0 (0.0)	
T1a (mi)	10 (0.7)	7 (0.6)	3 (0.8)	
T1a	15 (1.0)	14 (1.3)	1 (0.3)	
T1b	75 (5.0)	65 (5.8)	10 (2.6)	
T1c	86 (5.7)	66 (5.9)	20 (5.1)	
T2	64 (4.3)	45 (4.0)	19 (4.9)	
T2a	96 (6.4)	74 (6.6)	22 (5.7)	
T2b	82 (5.5)	58 (5.2)	24 (6.2)	
T3	315 (21.0)	235 (21.1)	80 (20.6)	
T4	730 (48.6)	532 (47.7)	198 (51.0)	
Missing	2 (0.1)	0 (0.0)	2 (0.5)	
N, *n* (%)				0.711
N0	172 (11.5)	130 (11.7)	42 (10.8)	
N1	115 (7.7)	89 (8.0)	26 (6.7)	
N2	874 (58.2)	641 (57.6)	233 (60.1)	
N3	331 (22.1)	245 (22.0)	86 (22.2)	
Nx	9 (0.6)	8 (0.7)	1 (0.3)	
M, *n* (%)				0.307
M0	1442 (99.0)	1066 (98.9)	376 (99.2)	
M1a	4 (0.3)	3 (0.3)	1 (0.3)	
M1b	2 (0.1)	1 (0.1)	1 (0.3)	
Mx	9 (0.6)	8 (0.7)	1 (0.3)	
Missing	48 (3.2)	37 (3.3)	11 (2.8)	
TNM stage, *n* (%)				0.077
IIIA T1a–c N2 M0	199 (13.2)	162 (14.5)	37 (9.5)	
IIIA T2a–b N2 M0	185 (12.3)	138 (12.4)	47 (12.0)	
IIIA T3 N1 M0	46 (3.1)	40 (3.6)	6 (1.5)	
IIIA T4 N0 M0	168 (11.2)	127 (11.4)	41 (10.5)	
IIIA T4 N1 M0	67 (4.4)	47 (4.2)	20 (5.1)	
IIIB T1a–c N3 M0	60 (4.0)	48 (4.3)	12 (3.1)	
IIIB T2a–b N3	58 (3.8)	40 (3.6)	18 (4.6)	
IIIB T3 N2 M0	185 (12.3)	129 (11.6)	56 (14.4)	
IIIB T4 N2 M0	355 (23.6)	255 (22.9)	100 (25.6)	
IIIC T3 N3 M0	66 (4.4)	44 (3.9)	22 (5.6)	
IIIC T4 N3 M0	110 (7.3)	82 (7.3)	28 (7.2)	

* Immunotherapy with durvalumab; Analyses performed with Stata/BE v17.0; M, metastasis; N, nodes; SD, standard deviation; T, tumor.

**Table 2:** Treatment.

	Total *N* = 1505	No Immunotherapy *N* = 1115	Immunotherapy * N = 390	
Intention of radiotherapy treatment				<0.001
Neoadjuvant radiotherapy or chemoradiotherapy, *n* (%)	22 (1.5)	18 (1.6)	4 (1.0)	
Sequential radical radiotherapy, *n* (%)	371 (24.7)	312 (28.0)	59 (15.1)	
Concurrent radical radiotherapy, *n* (%)	871 (57.9)	556 (49.9)	315 (80.8)	
Adjuvant postsurgical radiotherapy N2, *n* (%)	84 (5.6)	79 (7.1)	5 (1.3)	
Adjuvant postsurgical radiotherapy R1, *n* (%)	17 (1.1)	16 (1.4)	1 (0.3)	
Palliative radiotherapy, *n* (%)	20 (1.3)	18 (1.6)	2 (0.5)	
Exclusive radical radiotherapy, *n* (%)	76 (5.0)	76 (6.8)	0 (0.0)	
Unknown, *n* (%)	23 (1.5)	22 (2.0)	1 (0.3)	
Missing, *n* (%)	21 (1.4)	18 (1.6)	3 (0.8)	
Time on radiotherapy in days, median (IQR)	44.0 (40.0; 49.0)	44.0 (39.0; 49.0)	45.0 (42.0; 49.0)	0.521
Total dose of radiotherapy, mean (SD)	60.2 (7.5)	59.5 (8.2)	62.1 (4.7)	<0.001
Type of radiotherapy, *n* (%)				<0.001
3D	307 (20.4)	252 (22.6)	55 (14.1)	
IMRT/VMAT	1171 (77.8)	841 (75.4)	330 (84.6)	
Missing	27 (1.8)	22 (2.0)	5 (1.3)	
Control of IGRT with CBCT, *n* (%)				0.059
No	178 (11.8)	142 (12.7)	36 (9.2)	
Yes	1315 (87.4)	962 (86.3)	353 (90.5)	
Missing	12 (0.8)	11 (1.0)	1 (0.3)	
Number of chemotherapy cycles, mean (SD)	4.0 (2.1)	4.0 (2.3)	4.1 (1.7)	0.458
Scheme, *n* (%)				0.280
Platinum double	1107 (73.6)	788 (70.7)	319 (81.8)	
Others	248 (16.5)	185 (16.6)	63 (16.2)	
Missing	150 (10.0)	142 (12.7)	8 (2.1)	
Time on chemotherapy, median (IQR)	63.0 (42.0–77.0)	63.0 (43.0–79.0)	58.0 (42.0–76.0)	0.343
Surgery, *n* (%)	157 (10.4)	146 (13.1)	11 (2.8)	<0.001
Missing	16 (1.1)	12 (1.1)	4 (1.0)	
Intention, *n* (%)				<0.001
Radical	144 (9.6)	136 (12.2)	8 (2.1)	
Rescue	10 (0.7)	10 (0.9)	0 (0.0)	
Palliative/Biopsy	5 (0.3)	2 (0.2)	3 (0.8)	
Surgery type, *n* (%)				<0.001
Pneumonectomy	17 (1.1)	17 (1.5)	0 (0.0)	
Lobectomy	126 (8.4)	120 (10.8)	6 (1.5)	
Atypical resection	15 (1.0)	10 (0.9)	5 (1.3)	

* Immunotherapy with durvalumab; Analyses performed with Stata/BE v17.0; CBCT, cone-beam computed tomography; IGRT, Image guided radiotherapy; IMRT/VMAT, Intensity-Modulated Radiation Therapy/Volumetric Modulated Arc Therapy; IQR, interquartile range; SD, standard deviation.

**Table 3:** Progression pattern and metastasis localization in patients with concurrent radio-chemotherapy treatment.

	Total *N* = 871	No Immunotherapy *N* = 556	Immunotherapy * N = 315	
Relapse	459 (52.7)	314 (56.5)	145 (46.0)	0.008
Local	179 (20.6)	120 (21.6)	59 (18.7)	0.026
Local (within previous RT field)	116 (13.3)	78 (14.0)	38 (12.1)	0.11
Local (out of field of previous RT)	86 (9.9)	56 (10.1)	30 (9.5)	0.613
Distance	202 (23.2)	136 (24.5)	66 (21.0)	0.052
Local and distance	78 (9.0)	58 (10.4)	20 (6.3)	0.13
Local (within previous RT field)	57 (6.5)	42 (7.5)	15 (4.8)	0.376
Local (out of field of previous RT)	47 (5.4)	35 (6.3)	12 (3.8)	0.161
Metastasis				
Lung	68 (7.8)	54 (9.7)	14 (4.4)	0.036
Liver	44 (5.1)	33 (5.9)	11 (3.5)	0.053
CNS	94 (10.8)	66 (11.9)	28 (8.9)	0.829
Bone	81 (9.3)	56 (10.1)	25 (7.9)	0.944
Suprarenal	44 (5.1)	33 (5.9)	11 (3.5)	0.328
Lymph nodes	54 (6.2)	43 (7.7)	11 (3.5)	0.031
Others	25 (2.9)	14 (2.5)	11 (3.5)	0.883

* Immunotherapy with durvalumab; Analyses performed with Stata/BE v17.0; CNS, central nervous system; RT, radiotherapy.

**Table 4:** Survival rates according to immunotherapy treatment.

	Immunotherapy *N* = 315	No Immunotherapy *N* = 556
(a) Overall Survival		
6 months	96.0% (93.1%, 97.7%)	78.2% (74.5%, 81.5%)
12 months	89.0% (84.8%, 92.1%)	63.4% (59.1%, 67.4%)
24 months	73.6% (67.4%, 78.7%)	46.2% (41.5%, 50.8%)
(b) Progression-Free Survival		
6 months	88.1% (83.9%, 91.3%)	61.0% (56.7%, 65.0%)
12 months	70.2% (64.6%, 75.1%)	39.1% (34.9%, 43.4%)
24 months	45.4% (39.0%, 51.6%)	25.5% (21.6%, 29.6%)

**Table 5:** Overall survival.

	Univariable		Multivariable	
	HR (95% CI)	*p* Value	HR (95% CI)	*p* Value
Immunotherapy	0.42 (0.35, 0.52)	<0.001	0.42 (0.34, 0.52)	<0.001
Age	1.02 (1.01, 1.03)	<0.001	0.42 (0.34, 0.52)	<0.001
Gender				
Female	[reference]		[reference]	
Male	1.40 (1.16, 1.69)	<0.001	1.18 (0.97, 1.44)	0.093
TNM stage				
IIIA stage	[reference]		[reference]	
IIIB stage	1.16 (0.99, 1.36)	0.064	1.28 (1.09, 1.52)	0.003
IIIC stage	1.37 (1.09, 1.73)	0.008	1.48 (1.16, 1.88)	0.001
Histology				
Adenocarcinoma	[reference]		[reference]	
Epidermoid	1.45 (1.24, 1.70)	<0.001	1.29 (1.09, 1.53)	0.003
Others	1.49 (1.13, 1.95)	0.004	1.33 (1.01, 1.76)	0.040
Cardiovascular comorbidity	1.22 (1.05, 1.41)	0.010	1.04 (0.89, 1.22)	0.639
Respiratory comorbidity	1.22 (1.04, 1.42)	0.012	1.10 (0.93, 1.29)	0.260

Analyses performed with Stata/BE v17.0; CI, confidence interval; HR, hazard ratio.

**Table 6:** Progression-free survival.

	Univariable		Multivariable	
	HR (95% CI)	*p* Value	HR (95% CI)	*p* Value
Immunotherapy	0.51 (0.44, 0.59)	<0.001	0.48 (0.41, 0.57)	<0.001
Age	1.01 (1.00, 1.01)	0.049	1.00 (0.99, 1.01)	0.786
Gender				
Female	[reference]		[reference]	
Male	1.21 (1.04, 1.41)	0.013	1.14 (0.97, 1.34)	0.118
TNM stage				
IIIA stage	[reference]		[reference]	
IIIB stage	1.27 (1.11, 1.46)	<0.001	1.35 (1.17, 1.55)	<0.001
IIIC stage	1.40 (1.14, 1.72)	0.001	1.51 (1.22, 1.86)	<0.001
Histology				
Adenocarcinoma	[reference]		[reference]	
Epidermoid	1.17 (1.03, 1.34)	0.018	1.08 (0.94, 1.25)	0.270
Others	1.20 (0.94, 1.52)	0.140	1.10 (0.86, 1.40)	0.443
Cardiovascular comorbidity	1.20 (1.05, 1.36)	0.005	1.14 (0.99, 1.30)	0.058
Respiratory comorbidity	1.22 (1.07, 1.39)	0.003	1.19 (1.03, 1.36)	0.015

Analyses performed with Stata/BE v17.0; CI, confidence interval; HR, hazard ratio.

**Table 7:** Toxicity Grade > 2 in immunotherapy-treated patients.

Type of Toxicity	*N* (%)
Pneumonitis	24 (6.15)
Esophagitis	18 (4.6)
Intestinal	4 (1)
Hepatitis	4 (1)
Cutaneous	3 (0.7)
Thyroiditis	3 (0.7)
Nephritis	2 (0.5)
Cardiac	1 (0.25)
Neuropathy	1 (0.25)
Hematologic	1 (0.25)

Analyses performed with Stata/BE v17.0.

## Data Availability

Data are available from the corresponding author upon reasonable request.
